# Identification of Altered Evoked and Non-Evoked Responses in a Heterologous Mouse Model of Endometriosis-Associated Pain

**DOI:** 10.3390/biomedicines10020501

**Published:** 2022-02-21

**Authors:** Miguel A. Tejada, Ana I. Santos-Llamas, Lesley Escriva, Juan J. Tarin, Antonio Cano, Maria J. Fernández-Ramírez, Paulina Nunez-Badinez, Bianca De Leo, Philippa T. K. Saunders, Victor Vidal, Florent Barthas, Katy Vincent, Patrick J. Sweeney, Rowland R. Sillito, James Douglas Armstrong, Jens Nagel, Raúl Gomez

**Affiliations:** 1 Research Unit on Women’s Health-INCLIVA, Institute of Health Research, 46010 Valencia, Spain; mtejada@ugr.es (M.A.T.); anais_santos91@yahoo.es (A.I.S.-L.); lesleyescrivaardid@gmail.com (L.E.); juan.j.tarin@uv.es (J.J.T.); antonio.cano@uv.es (A.C.); 2 Department of Cellular Biology, Functional Biology and Physical Anthropology, University of Valencia, 46100 Burjassot, Spain; 3 Department of Pediatrics and Obstetrics and Gynecology, University of Valencia, 46010 Valencia, Spain; mafernanrami@hotmail.com; 4 Department of Obstetrics and Gynecology, Hospital Clínico Universitario, 46010 Valencia, Spain; 5 Bayer AG. Research & Early Development, Pharmaceuticals, Reproductive Health, Müllerstr. 178, 13342 Berlin, Germany; paulina.nunez-badinez@bayer.com (P.N.-B.); bianca.deleo@bayer.com (B.D.L.); 6 Centre for Inflammation Research, Queen’s Medical Research Institute, The University of Edinburgh, 47 Little France Crescent, Edinburgh EH16 4TJ, UK; p.saunders@ed.ac.uk; 7 Faculty of Science, International University of La Rioja, Avda de la paz 137, 26006 Logrono, Spain; victor.vidal@unir.net; 8 Grünenthal GmbH, 52099 Aachen, Germany; florent.barthas@grunenthal.com; 9 Nuffield Department of Women’s and Reproductive Health, University of Oxford, Oxford OX1 2JD, UK; katy.vincent@wrh.ox.ac.uk; 10 Actual Analytics, 99 Giles Street, Edinburgh EH6 6BZ, UK; psweeney@actualanalytics.com (P.J.S.); rsillito@actualanalytics.com (R.R.S.); douglas.armstrong@ed.ac.uk (J.D.A.); 11 School of Informatics, University of Edinburgh, 10 Crichton Street, Edinburgh EH8 9AB, UK; 12 Bayer AG. Research & Early Development, Pharmaceuticals, Exploratory Pathobiology, Aprather Weg 18a, 42096 Wuppertal, Germany; jens.nagel@bayer.com; 13 Department of Pathology, University of Valencia, 46010 Valencia, Spain

**Keywords:** endometriosis, heterologous model, pain, evoked and non-evoked response

## Abstract

The aim of this study was to develop and refine a heterologous mouse model of endometriosis-associated pain in which non-evoked responses, more relevant to the patient experience, were evaluated. Immunodeficient female mice (N = 24) were each implanted with four endometriotic human lesions (N = 12) or control tissue fat (N = 12) on the abdominal wall using tissue glue. Evoked pain responses were measured biweekly using von Frey filaments. Non-evoked responses were recorded weekly for 8 weeks using a home cage analysis (*HCA*). Endpoints were distance traveled, social proximity, time spent in the center vs. outer areas of the cage, drinking, and climbing. Significant differences between groups for von Frey response, climbing, and drinking were detected on days 14, 21, and 35 post implanting surgery, respectively, and sustained for the duration of the experiment. In conclusion, a heterologous mouse model of endometriosis-associated evoked a non-evoked pain was developed to improve the relevance of preclinical models to patient experience as a platform for drug testing.

## 1. Introduction

Endometriosis is estimated to affect 10% of women of reproductive age [1,2,3,4,5]. Chronic pain is a disabling symptom reported in approximately 50–60% of affected women [2] and has a high impact on the quality of life of the patients [6,7,8,9] as well as high socioeconomic cost including health care expenses [10,11,12]. Endometriosis patients are often treated with repeated surgeries, and no current medical treatment is able to reliably alleviate chronic pelvic pain. Many drugs, when administered long-term, have unacceptable side effects [4].

In the quest to develop new and effective treatments, a number of preclinical models of endometriosis have been developed and used to both explore the etiology of pain mechanisms and to facilitate the development of drugs for treatment. The use of primates is hampered by economic and ethical considerations making rodents a more feasible option for this purpose. In practice, however, promising preclinical data in rodent models are often not reproduced in clinical trials, leading to increased attrition rates in the drug discovery pipeline. This apparent problem in translation from rodent models may be due to (1) the inability to fully model the complex physiology of human endometriosis (construct validity) and/or (2) limitations in the ability to use reliable and reproducible behavioral measures as a surrogate measure of ‘pain’ in such models (face validity).

In regard to construct validity, the main limitation arises from the fact that rodents do not menstruate and therefore cannot develop spontaneous endometriosis [13]. Murine “lesions” consisting of psedudodecidualized tissue can be generated and implanted in recipients to mimic the “menstruating” phenotype of endometriosis in a homologous model. However, still, it can be questioned whether such murine donor tissue recapitulates the pathophysiology of endometriotic lesions. In this context, heterologous models, although not free of limitations [14], allow to focus on the pathophysiology of the disease by using lesions recovered from patients as a source of donor material. However, most, if not all, of the studies assessing pain in these models have been conducted in the homologous animal setting.

With regard to face validity, the challenges of reliably assessing pain in rodent models have been reviewed [15,16]. As pain perception cannot be reported by rodents, surrogate measures based on altered behavior (i.e., non-evoked responses) and/or altered response to stimuli (evoked responses) are used [17,18,19]. Although pain experienced by endometriosis patients may be exacerbated by physical stimuli (i.e., dysuria, dyspareunia dyschezia) [2,20], much of the chronic pain suffered by patients may not be directly tied to an obvious mechanical stimulus. With this background, it is likely that approaches to testing pain in rodent models based on non-evoked responses might be more relevant to the patient experience and useful for translational studies.

Given the potential advantages mentioned above, we reasoned that it would be timely to explore whether a heterologous model of endometriosis-associated pain could be developed and refined. For this purpose, mice were implanted with human endometriosis tissue lesions, and their reactions were explored using both evoked and non-evoked behavioral responses.

## 2. Materials and Methods

### 2.1. Experimental Design

A total of 24, 8–10-week-old female B6.129S-Rag2tm1Fwa Cd47tm1Fpl Il2rgtm1Wjl/J (TKO) immunodeficient mice (The Jackson Laboratory, Bar Harbor, ME, USA) were used for the study. Due to their CD47 deficiency, this strain of mice is resistant to the onset of Graft Versus Host Disease. All animals were housed in specific pathogen-free conditions at 26 °C with a 12 h light/12 h dark cycle and fed ad libitum. The study was approved by the Institutional Animal Care Committee at the University of Valencia (2020/VSC/PEA/0135), and all procedures were performed following the guidelines for the care and use of mammals from the National Institutes of Health.

**Figure 1:**
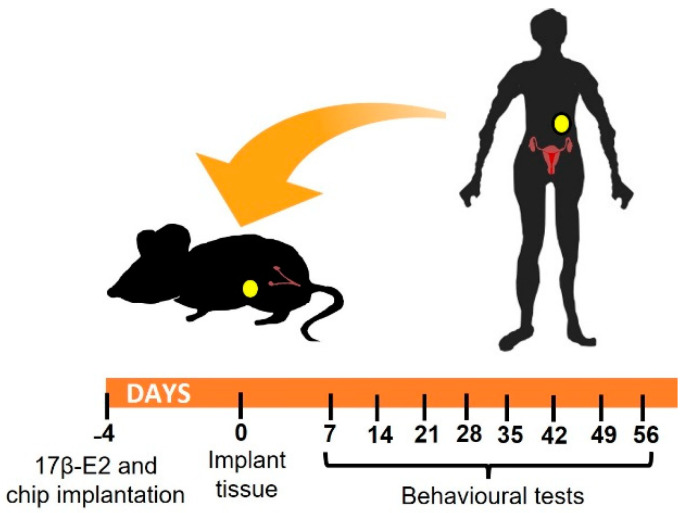
Experimental design: image shows schematic representation of timeline. Recipients that were 6–8-week-old immunocompromised animals were implanted with a 17β-E2 pellet in the neck and, if required, a microtracker chip in the groin. 4 days later, mice underwent surgery to place human tissue (endometriotic lesions or fat) in the peritoneal cavity. Behavioral non-evoked and evoked pain responses were monitored every week starting on day 7 after surgery.

A summary of the experimental design is given in Figure 1. Briefly, mice were randomly assigned to experimental or control groups to be xenografted with 4 human endometriotic lesions (N = 12) or fat (N = 12) respectively on the inner peritoneal wall in the abdominal area. Four days before xenograft surgery, all mice were given a 17β-E2 oestradiol pellet. In each group, half of the animals (N = 6) were employed for assessment of evoked pain responses to von Frey filaments on the abdominal wall and hind paw. In addition, nesting behaviors were also assessed as a representative measure of a non-evoked test requiring minimal operator manipulation. The remaining animals (N = 6) in each group were implanted with a small transponder chip in the left groin and left undisturbed in their cages for automated monitoring of non-evoked responses using an in cage analysis system (Actual Analytics Ltd., Edinburgh, UK; detailed procedures described below). To minimize human intervention, animals were assessed weekly for non-evoked responses, while evoked responses were assessed every two weeks (with the exception of the initial week). The time course of the experiment was 8 weeks (56 days) from the time of model induction (i.e., lesion implant surgery). At the end of the experiment, mice were sacrificed, and lesions were excised for histological analysis and immunofluorescent detection of nerve fibers.

### 2.2. Heterologous Mouse Model of Endometriosis

#### 2.2.1. Preparation of Endometriosis Recipient Mice: Oestradiol Pellet and Chip Implantation

Four days in advance of surgery to implant human tissue, all mice (N = 24) received 60-day-release capsules containing 18 mg of 17β-E2 (Innovative Research of America, Sarasota, FL, USA) placed under the neck skin to stimulate survival and growth of lesions. Additionally, during the same procedure, a standard ISO 2 × 12 mm pre-packed and sterile RIFD tag microchip (PeddyMark, Elsenham, UK) was inserted subcutaneously into the groin area of mice intended for *HCA*-monitoring (N = 12). During these procedures, mice were briefly anesthetized with isoflurane 2% (Abbott Laboratories, Queenborough, UK).

#### 2.2.2. Obtention and Preprocessing of Human Endometrial Biopsies

The use of human tissue specimens was approved by the Institutional Review Board and Ethics Committee of INCLIVA (2017-253, 2017). Human ectopic endometrial tissue and fat were acquired from 2 patients undergoing laparoscopy surgery to remove endometriotic lesions. Peritoneal and endometrioma lesions (both types of lesions present in both patients), as well as small pieces of periovarian fat, were carefully excised during surgery and selected by their visual appearance (i.e., free of apparent necrosis or burns) for subsequent processing. “Fresh” tissues were placed immediately into maintenance M199 medium containing 10% fetal bovine serum and antibiotic-antimycotic solution (Gibco, New York, NY, USA) and transported on ice to the laboratory. Fragments from each patient were placed on a 10 cm Petri dish, cut into 3–5 mm^3^ pieces with a scalpel, and placed on ice, ready to be implanted in half of the animals of each subgroup. A small number of pre-processed tissue fragments were fixed in PFA 4% for preimplantation histological analysis.

#### 2.2.3. Endometrial Fragment Implantation

Mice were injected with buprenorphine 5 mg/kg (s.c) and anesthetized with isoflurane 2% (Abbott Laboratories, Queenborough, UK). A small incision was made in the abdominal area with sharp scissors to access the peritoneal cavity. Subsequently, 4 pieces of human tissues consisting either of lesions (usually two pieces of endometrioma and two pieces of peritoneal lesions) or fat (control) per mouse were glued on the inner face of the peritoneal wall with biological tissue glue (Vetbond™ Tissue Adhesive, 3M, Fisher Scientific, Madrid, Spain). The muscular and skin incisions were closed with absorbable 6/0 sutures, as was previously described by our group [21,22].

### 2.3. Behavioral Tests

#### 2.3.1. Mechanical Hypersensitivity

A von Frey test was used to evaluate the mechanical pain thresholds. The animals were acclimated for 2 h in a methacrylate compartment (8 cm long × 8 cm wide × 12 cm high) placed on an elevated mesh platform. Hyperalgesia was determined by measuring the withdrawal response to a punctate mechanical stimulation (von Frey hair filaments 0.02–2 g force, Stoelting, Dublin, Ireland) of the abdomen and hind paw using the up–down paradigm [23] starting with the 0.6 g filament. Filaments were applied twice for 2–3 s, between-application intervals of at least 30 s to avoid sensitization to mechanical stimulation. The response was considered positive if the mice immediately showed licking, biting, flinching, or rapid withdrawal of the stimulated area.

#### 2.3.2. Nesting

**Figure 2:**
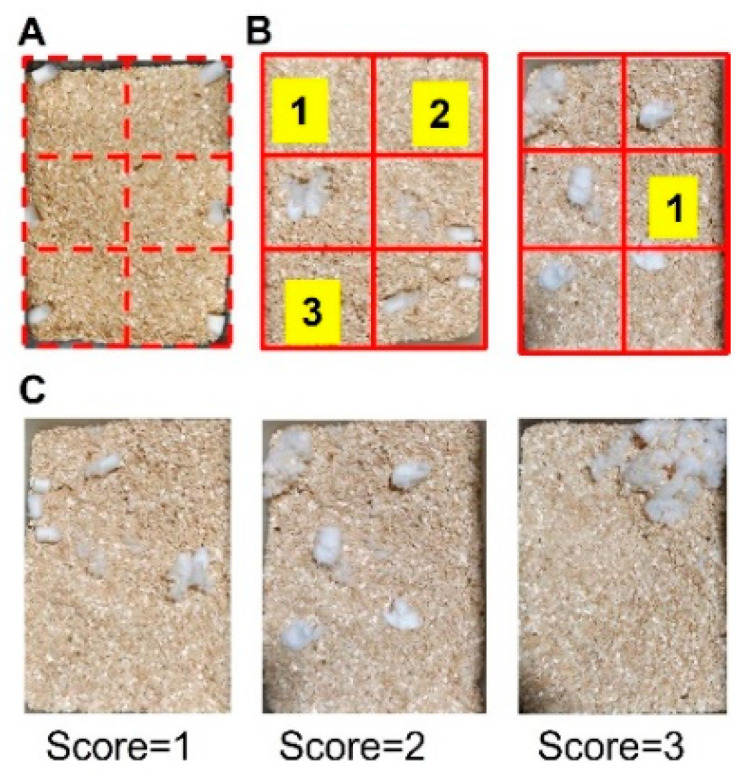
Schematic image of nesting test. (**A**) Example of a cage divided into 6 equivalent areas in which a piece of cotton is placed in each area. After 3 h, the total number of cleared spaces was counted (**B**). Illustrative examples of different nest quality scores: 1 = cotton not broken, 2 = cotton partially broken, 3 = cotton totally broken (**C**).

To perform nesting analysis, a cage was virtually divided into 6 areas of equivalent space, and six pieces of cotton were placed, one in the center of each of the areas (Figure 2A). Subsequently, mice were placed in the designated cage for 3 h, and their ability to build the nest was evaluated by the operator using two parameters: (1) number of empty areas based on Negus 2015 [24] and (2) quality of the nest based on Deacon 2006 [25] with brief modifications. Areas were considered empty if they were clear of cotton material (see Figure 2B for illustrative examples). The quality of the nest was coded according to the following scale: 1 = cotton ball untouched/not broken, 2 = cotton ball partially broken, 3 = cotton was totally broken (see Figure 2C for illustrative examples).

#### 2.3.3. Home Cage Activity (*HCA*)

The Home Cage Analyzer (*HCA*; Actual Analytics Ltd., Edinburgh, UK, https://www.actualanalytics.com/products (accessed on 16 February 2022)) is an instrument that allows continuous automated monitoring of individual animal behavior in groups of mice. The system contains an array of 18 detectors beneath the cage, which are capable of detecting the RIFD trackers inserted in the mice to monitor locomotor activity. The list of parameters measured includes distance traveled, time spent in the center vs. outer regions of the cage, and social proximity to cage-mates. The system is also equipped with a video camera that allows collecting near-infrared (NIR) footage. Additional behaviors automatically measured from the NIR video included time spent drinking and climbing [26]. Mice were inserted with RFID tags 4 days before surgical model induction. Once lesions had been placed, mice were put back on their cages (N = 3 mice per cage) and left undisturbed for the whole experiment. *HCA* recordings were performed weekly for 48 consecutive hours over a period of 8 weeks. Actual *HCA* Analyser™ software was used to analyze the raw data files generated by the *HCA* and to download the behavior results into a spreadsheet for further analysis.

### 2.4. Histology and Immunofluorescence

For histological purposes, lesion fragments collected preimplantation as well as xenografted lesions collected from mice at the end of the experimental run (i.e., 56 days after implantation) were fixed in 4% PFA overnight at 4 °C, before being embedded in paraffin wax, and slices cut at 5 μm. Tissue sections were placed on slides, deparaffinized in xylol, rehydrated in decreasing concentrations of alcohol, and distilled water before being stained with haematoxylin and mounted for analysis by a pathologist.

For immunofluorescent analysis of nerve fibers, lesion tissue fragments pre and post implantation were fixed with PFA 4% and subsequently cryoprotected in 30% sucrose phosphate buffer for 48 h. Afterward, samples were frozen in OCT embedding compound, cut into sections of 20 μm width using a cryostat, and incubated with anti-β-III tubulin (1:100, PA5-85639, Thermo Fisher Scientific, Waltham, MA, USA) primary antibody, overnight at 4 °C. The next day, sections were incubated with a secondary goat anti-rabbit Alexa 594 (1:500, A11012 Thermo Fisher Scientific) antibody for 1 h at room temperature, labeled with DAPI (1:1000, Thermo Fisher Scientific) for 5 min, and coverslips were added together with antifade mounting medium (Vector Laboratories, Burlingame, CA, USA) for visualization.

### 2.5. Statistical Analysis

Data were expressed as mean ± SEM. Data were analyzed with the SigmaPlot 12.0 program (Systat Software Inc., San Jose, CA, USA). A one-way ANOVA followed by Student Newman–Keuls post-hoc test was used to discern the effects of the lesion implant vs. control group in each time point evaluated. *p* < 0.05 was considered statistically significant.

## 3. Results

### 3.1. Mice with Human Endometriotic Lesions Present with Decreased Abdominal and Hind Paw Thresholds in Von Frey Tests

**Figure 3:**
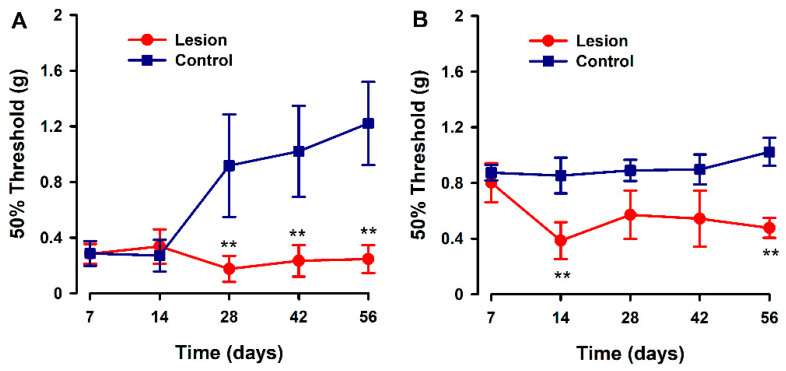
Evaluation of abdominal and hind paw mechanical pain thresholds in a heterologous model of endometriosis. Graphs show abdominal (**A**) and hind paw (**B**) mechanical threshold in animals implanted with human endometriotic lesion (red line) or fat tissue (blue line) at several time points after implanting surgery. Data in each time point are expressed as mean ± SEM values of each group (N = 6 per group). A one-way repeated ANOVA followed by Student Newman–Keuls post-hoc test was performed to analyze comparisons between groups. ** *p* < 0.01 = statistically significant differences between lesion and control groups at each time point.

We evaluated the development of hyperalgesia to a mechanical stimulus in the hind paw and abdominal area during a time course after human tissue implantation (endometriotic lesion or fat tissue) in recipient mice. Overall, significant decreases in pain thresholds to mechanical stimulation (increased sensitivity) were detected in the experimental versus control groups, from 14 to 28 days post surgery, and were sustained for the duration of the experiment. The pattern of response to the von Frey test was different depending on the test site. On the abdomen, mechanical pain threshold values started low in both groups, and control animals increased their threshold values starting 28 days after model induction; while in the experimental group, these thresholds were consistently low over the time course and up to 56 days after model induction (Figure 3A). In contrast, mechanical pain evaluation at the hind-paws revealed higher pain thresholds at day 7 for both groups, but in control animals, these thresholds remained high at all times while in lesion-implanted mice, thresholds abruptly decreased on day 14 and remained low for the rest of the observation period (Figure 3B).

### 3.2. Nesting Behaviors Remained Unchanged in the Heterologous Model

**Figure 4:**
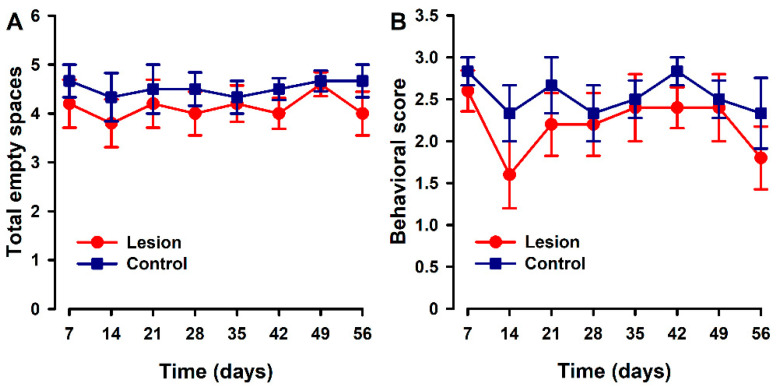
Nest building time course in animals with endometriosis or its control. (**A**) Nest building was evaluated by counting the number of cleared areas or (**B**) with a quality score (1 = intact bedding, 2 = bedding partially broken, 3 = bedding totally broken). Lesion and control groups are represented by red and blue lines. Data in each time point express mean ± SEM values of each group (N = 6 per group). A one-way repeated ANOVA followed by Student Newman–Keuls post-hoc test was performed to analyze comparisons between groups. Statistically significant differences between groups were not detected.

Parameters associated with nesting were quite stable over the time course in both groups. Mice implanted with lesions showed a trend to a slight decrease in the number of cleared zones (Figure 4A) and on nest quality (Figure 4B) with respect to the control group at some specific time points. However, no statistically significant differences were found between groups at any time point.

### 3.3. Mice with Human Endometriotic Lesions Present with Decreased Climbing and Drinking during Home Cage Analysis (HCA)

**Figure 5:**
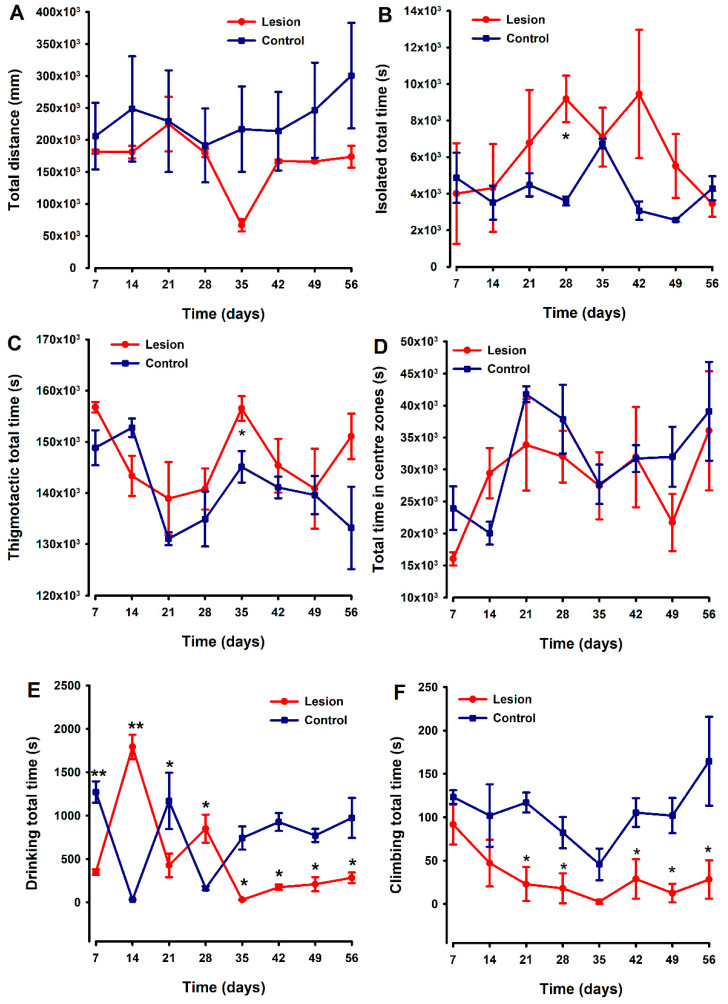
Comparison of temporal pattern of home cage activity between animals implanted with endometriosis lesions or fat. Parameters analyzed were distance traveled (**A**), time isolated (**B**), time spent in centers zone (**C**), thigmotactic (**D**), and the time spent climbing (**E**) and drinking (**F**). The lesion and control groups are represented by the red and blue lines, respectively. Each point and vertical line represent the mean ± SEM of the values obtained of 6 animals. A one-way repeated ANOVA followed by Student Newman–Keuls post-hoc test was performed to analyze comparisons between groups during the time course. * *p* < 0.05, ** *p* < 0.01 = Statistically significant differences between lesion and control groups at each time point.

Non-evoked behavioral tests were simultaneously recorded and analyzed using the *HCA* equipment. Locomotion was evaluated as “distance travelled” by each mouse during 48 h recordings performed weekly over 8 weeks, with no differences between groups detected (Figure 5A). Mice with endometriosis tissue implants tended to spend more time isolated and shown thigmotactic behavior (i.e., they spent more time near the edges of the cage compared to control (fat implanted) animals (Figure 5B,C)), with statistically significant differences being detected at two specific time points (Figure 5B and Figure 5C, respectively) in each case. However, overall in these small groups, the differences in behaviors were quite variable, and any clear trend or strong differences between groups sustained over the duration of this experiment could be detected. Regarding the parameter “time in centre zones”, lesion and control groups showed a similar temporal course without significant differences between both (Figure 5D).

The drinking patterns of the animals exhibited some behavioral variability between groups during the first 28 days of the experiment. However, thereafter (day 35), differences emerged with endometriosis-lesion-containing mice spending significantly less time drinking than controls, and this was maintained until the end of the experiment (Figure 5E). Lesion-implanted animals also spent significantly less time climbing compared to control animals starting on day 21. The significant decrease in climbing behavior in the experimental group remained until the end of the evaluation period, with statistically significant differences against the control group detected at most of the time points evaluated (Figure 5F).

### 3.4. Post-Implantation Lesions Preserve the Histological Architecture and Nerve Fiber Density of Human Lesions at Time of Surgery

**Figure 6:**
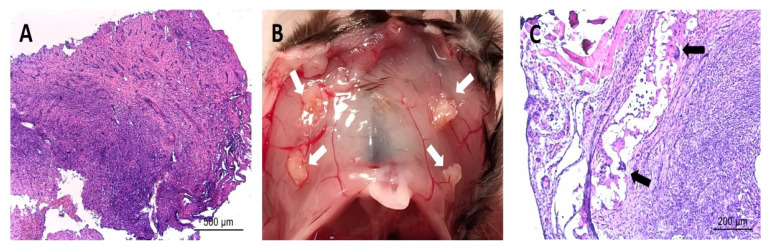
Image in (**A**) shows representative histological section of an endometriotic lesion recovered from human patients before being implanted in mice. Image in (**B**) shows representative observation of the macroscopic appearance of lesions (white arrows) recovered from mice at sacrifice at the end of the study period. Image in (**C**) shows representative histological section of an endometriotic lesion recovered from mice at the end of the study period. Note the parallels in the overall tissue architecture between the fresh and implanted tissues in regards to dense stromal tissue and few/absent glands. Note also detail of presence of immune cell infiltrate (i.e., giant cells, black arrows) in implanted tissue. Scale bar is represented in histological images.

**Figure 7:**
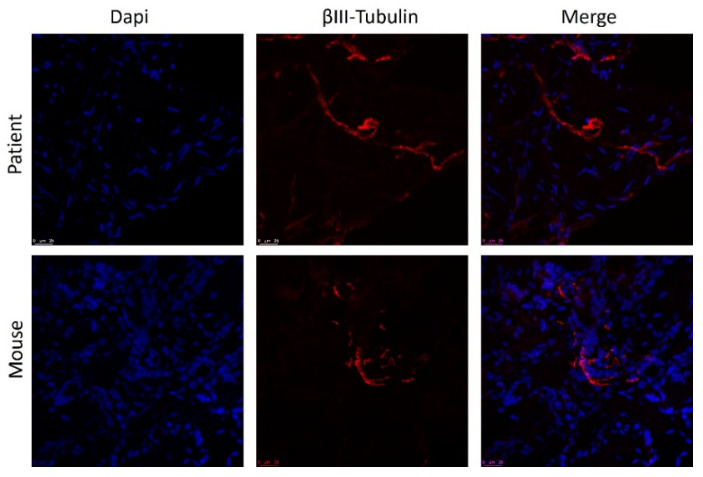
Staining of nerve fibers in human endometrial lesion before and after implantation in mice. Nuclei are stained with DAPI (blue) and fibers with anti-Beta III tubulin (red). Scale bar = 25 µm.

Overall, the histologic appearance of endometriotic tissue preimplantation was in agreement with the pattern expected from their anatomical location in the patients. Specifically, endometrioma (ovarian) lesions were characterized by a dense stromal tissue devoid of glands, and at some points, foci of ovarian tissue adjoining the abnormal areas with the presence of primary and primordial follicles was detected. In superficial peritoneal lesions, most of the tissue sections were characterized by abundant dense stromal tissue devoid of glands (Figure 6A). A minor number of sections presented small occluded glands with sparse and sporadic distribution. The tissue was moderately innervated with frequent nerve fibers detected using immunostaining for beta III tubulin (Figure 7). Lesions recovered from animals did not show any significant alteration of the tissue architecture when compared to their preimplantation counterparts (Figure 6C). Lesions from endometrioma origin retained their dense stromal tissue with no glands, and lesions of peritoneal origin were small and barely detectable, thus in agreement with observations in preimplantation tissue. In some lesions, apparent foci of inflammation/tissue remodeling were revealed by the presence of macrophages in the form of giant cells in the peripheral zones of the endometriotic tissues (Figure 6C). The extent of innervation was also similar after the implanting period, and no obvious changes in density/frequency were observed (Figure 7).

## 4. Discussion

Overall, pain is the main endpoint assessed in endometriosis clinical trials in agreement with authorities (i.e., FDA, EMA) requirements for drug approval for such indication. Therefore a great effort is being taken by academic and pharmaceutical entities to develop a reliable animal model for preclinical testing of endometriosis-associated pain. Setting up animal models providing clinical transferability requires a deep knowledge of endometriosis physiopathology, which in turn demands appropriate models for research. A potential way to overcome this vicious circle picture is to empirically mimic endometriosis conditions in models in the belief that this will reflect the human disease physiology and, as a consequence, associated symptomatology. With such an approach herein, we attempted to establish and validate a new heterologous mouse model of endometriosis-associated pain.

Our results revealed differences in evoked and non-evoked pain-associated behaviors that were affected by the implantation of endometriotic lesions in comparison to a control tissue (fat). Specifically, significant differences between groups were found in the mechanical pain thresholds on the abdomen and hind paw (evoked) as well as in drinking and climbing behaviors (non-evoked) that were sustained for the duration of the experiment.

In regard to evoked manifestations, the pattern of response to the von Frey test was different depending on the test site. In the hind paw, the increased sensitivity in the experimental animals was observed starting on day 14 post implantation. The timing is consistent with reports from others in the homologous model [27,28] and agrees with the period required for neuroangiogenesis to take place in lesions [27,28,29,30]. Central [31] and/or cross-organ sensitization [32] are likely mechanisms to explain the altered responses to von Frey stimulus in hind paw provided its anatomical distance to the xenografts tissue. Notably, this mechanism was not activated in the controls with fat tissue implants. The Wang group [33] also used fat as a control tissue in their rat model of endometriosis and showed that expression of the TRPV1 channel was increased in the dorsal root ganglia adjacent to endometrial tissue lesions but not those formed from fat, providing a plausible mechanism by which pain pathways are altered by endometriosis lesions that would be in agreement with our results. In contrast to hind-paws, abdominal von Frey threshold values were low since the beginning of the time course, and differences between groups became evident 28 days after lesion implantation. Similar experiences in the homologous model have been reported; however, lowered abdominal von Frey thresholds in endometriosis vs sham-operated animals as early as one week post implantation [34]. Provided that xenografted tissue and surgery scars are anatomically close to the abdominal sites where von Frey filaments are applied, a potential explanation for divergent findings between studies might be as follows: Surgery and or tissue implantation resulted in an inflammatory insult masking/overlapping/mimicking pain due to peripheral nerve sensitization in endometriosis lesions. As long as mice recovered from the inflammatory insult, abdominal von Frey thresholds would rise to normal values in the controls while maintained low in the endometriosis animals in whom peripheral sensitization persisted.

In regard to non-evoked responses, drinking and climbing behaviors showed significant differences between test and control animals sustained over time. While this is the first time that such behaviors are reported to be altered in a mouse model of endometriosis, the same parameters have been used to identify non-mechanical-associated pain in mouse models of neuropathy and cancer disease [35,36]. It is of note that both climbing and drinking showed sustained alterations starting 21 and 35 days after lesion implantation, respectively. These observations are in agreement again with the idea that alteration of non-evoked responses (i.e., “chronic pain”) starts to be detected once lesions are established in the recipient animals [29] but not at earlier stages [37].

Neuroangiogenesis has been suggested to play a key role in the onset of pain [30], but this statement might be somehow simplistic attending to clinical observations. In this regard, vessel density does not seem to be related to pain experience as reflected by the poor vascularization of deep infiltration lesions (DIE), which tend to provoke the most painful experiences [38]. Indeed, even among DIE lesions, those with higher vascularization have not been linked with higher scores in patient pain questionaries [39]. A more accepted consensus relies on the fact that pain tends to be exacerbated by perineural and immune cell infiltration [38,40]. In a similar fashion, we speculate that evoked and non-evoked altered responses in our model arise from inflammation and sensitization of innervated lesions. Indeed, we observed nerve fibers as well as infiltration of immune cells inside the lesions in our model. We have to agree, however, that ours is mostly an exploratory study, and further analysis of the factors influencing evoked and non-evoked responses will be required to validate the model. Another weakness of our model is that the immune system of recipient animals is defective on the lymphoid lineage. So, although monocytes [41] and mast cells [38], which play an important n role in endometriosis-related inflammatory pain [42], are present, their interaction with the adaptive system is not preserved.

Overall, the major strengths of this study rely on the efforts to enhance face and construct validity of this newly developed animal model. In order to increase the construct validity, ectopic lesions rather than eutopic endometrium were used as the source of donor material, thus better reflecting the pathophysiology of disease. In an attempt to improve face validity (i.e., for a better characterization of pain-associated behavior), we combined both evoked and non-evoked measures in the model with special attention to the latter [43]. Non-evoked tests are not free of limitations with acknowledged challenges in their execution and in the unbiased interpretation of results. For example, behavioral responses may be assessed in an unnatural environment (i.e., animals are isolated, tested out of the cage, social interactions are neglected) involving human contact, which may induce stress, thus increasing the risk of bias and decreasing the quality of the recorded data. To circumvent this limitation, we used an *HCA* in cage system, which allowed a noninvasive way of observing rodents in their home cages, thereby preserving social interactions and avoiding operator bias [26,44].

An additional advantage of our model is the use of biological tissue glue for implanting lesions. Such a procedure is compatible with appropriate construct validity and is very helpful for the recovery of precious implanted tissue at the end of the experiment. In regard to the former, the widely used rat model described by Berkley et al. in their seminal paper [45] uses a stitch to position tissue on the mesentery. Stitch models injury the host recipient tissue and require the use of full-thickness uterus as donor material so that they can put the stitch through myometrium, which obviously is not a physiological replication of the human disease. In regards to the latter, while direct injection of the endometrium into the peritoneum [46] may model retrograde menstruation, in that model, the lesions form randomly and with a low percentage of the tissue attaching/surviving. In our view thus, the use of biological tissue glue should be considered as a useful refinement of the current homologous and heterologous rodent models of endometriosis.

Overall, there are a couple of findings from this study that seem to arise as “policymakers” for subsequent exploration/better characterization of animals models of endometriosis-associated pain in the future.

First, we would like to point on the convenience of combining evoked and non-evoked tests in the analysis of pain responses. In this regard, recording of climbing time and the von Frey test might be a useful example of how a combination of evoked and non-evoked tests can be used to allow a better characterization of pain in the endometriosis models [47]. Although highly speculative, the drastic and erratic changes in drinking during the first 3–4 weeks might merely reflect disturbance/discomfort associated with surgery, which otherwise could not have been properly identified without the use of the von Frey test in the abdomen.

Second, we would like to claim attention to the need/convenience to assure/extend lesion survival to allow identification and follow-up of the whole plethora of “pain” responses. A clear example of the above is the non-evoked climbing parameter, which became “unmasked” after 35 days post implantation surgery. In this context, one of the questions arising from our findings is whether the administration of exogenous oestradiol that we used to promote lesion survival might compromise the translatability of the model. A recent study comparing the characteristics of different homologous models suggests that, in the absence of oestradiol supplementation, lesion survival might not be consistently maintained beyond 21–28 days [42]. As our study results suggest, at least 3–4 weeks are required to initiate the full range of behaviors associated with pain, suggesting it is an important supplement. To our knowledge, there have not been any other studies analyzing the effects of oestradiol on lesion survival in the heterologous mouse model [48] beyond day 21 days post implantation, and our results are therefore novel. To assess the effects of lesion survival and the translational relevance of our model, a next logical step for further evaluation will be to determine the minimal dose of oestradiol required to support tissue survival and the alteration of pain-associated behaviors over a sufficient sustained period of time after model induction.

## 5. Conclusions

While a range of preclinical homologous animal models have been used to better understand the etiology of endometriosis-associated pain, non-evoked tests for characterization of pain-like behaviors have been rarely used [34,49,50,51]. There has not been a single report (to our knowledge) employing heterologous models for such purpose and no apparent reason for such asymmetry in their use.

In conclusion, in this study, we have developed, for the first time, a heterologous mouse model of endometriosis-associated pain in which alteration of both evoked and non-evoked behaviors can be identified starting 14–28 days after lesion implantation and are sustained for 56 days. After further validation and refinement, we hope this model will provide a platform for testing potential medical therapies for endometriosis-associated pain and improve translation for patient benefit.

## Data Availability

The data presented in this study are available on reasonable request from the corresponding author.
